# Robotic-assisted laparoscopic repair of isthmoceles: the feasibility of operative treatment and recommendations for patient selection

**DOI:** 10.1177/26334941261426108

**Published:** 2026-03-04

**Authors:** Sa’ed Almasarweh, Rainer Kimmig, Anna Magdalena Jakob, Anika Hüser, Paul Buderath, Roland Csorba, Angela Köninger, Antonella Iannaccone

**Affiliations:** Department of Obstetrics and Gynaecology, Essen University Hospital, Hufelandstraße 55, D-45147 Essen, Germany; Department of Obstetrics and Gynaecology, Essen University Hospital, Essen, Germany; Department of Obstetrics and Gynaecology, Essen University Hospital, Essen, Germany; Department of Obstetrics and Gynaecology, Essen University Hospital, Essen, Germany; Department of Obstetrics and Gynaecology, Essen University Hospital, Essen, Germany; Department of Obstetrics and Gynaecology, Essen University Hospital, Essen, Germany; Department of Obstetrics and Gynaecology, Krankenhaus Barmherzige Brüder Regensburg - Klinik St. Hedwig, Regensburg, Germany; Department of Obstetrics and Gynaecology, Essen University Hospital, Essen, Germany

**Keywords:** healing ratio (HR), isthmoceles, residual myometrial thickness (RMT), robotic-assisted laparoscopic repair, uterine niche

## Abstract

**Background:**

Uterine niches (isthmoceles) are myometrial defects commonly occurring after caesarean sections and may lead to abnormal uterine bleeding, pelvic pain and impaired fertility. Robotic-assisted surgical approaches have emerged as a minimally invasive option for their management, yet data on their efficacy remain limited.

**Objective:**

To evaluate the efficacy of robotic-assisted laparoscopic repair of uterine niches in improving clinical symptoms and fertility outcomes.

**Design:**

A retrospective observational study conducted at a tertiary university hospital.

**Methods:**

All patients who underwent robotic-assisted laparoscopic repair of uterine niches between 2013 and 2023 were included. Preoperative assessments included transvaginal ultrasound and hysterosalpingo-ultrasonography to evaluate residual myometrial thickness (RMT) and niche morphology. The surgical procedure entailed isthmocele resection followed by double-layer myometrial suturing using the Da Vinci Robotic System®. Pre- and postoperative RMT measurements were compared to assess myometrial restoration. Symptom resolution and postoperative fertility outcomes were also evaluated.

**Results:**

Fifty-one patients underwent robotic-assisted laparoscopic repair during the study period. The mean RMT significantly increased from 1.75 ± 1.4 mm preoperatively to 4.9 ± 3.4 mm postoperatively (*p* < 0.001). While niche diameter reduction was not statistically significant, the healing ratio demonstrated a significant improvement (*p* < 0.001). Among symptomatic patients with postoperative symptom assessment (*n* = 28), 20 (71.4%) reported complete or partial symptom resolution. Among patients desiring conception postoperatively (*n* = 36), 26 conceived; among pregnancies, 17/26 (65.4%) resulted in live birth, 1/26 (3.8%) was ongoing at last follow-up and 3/26 (11.5%) had an unknown outcome.

**Conclusion:**

Robotic-assisted laparoscopic repair was associated with improvement in symptoms related to uterine niches. The technique significantly improves myometrial thickness and supports favourable reproductive outcomes. Further prospective studies are warranted to establish standardised treatment guidelines and assess long-term efficacy.

**Trial registration:**

Not applicable.

## Plain language summary

Feasibility of robotic treatment of isthmoceles

This study looked at how effective robotic-assisted surgery is for treating uterine niche (also known as an isthmocele), which can cause symptoms like pelvic pain, abnormal bleeding, and difficulty getting pregnant. These niches often form after a cesarean section and can affect the thickness of the uterine wall. The researchers reviewed records from 51 women who had robotic-assisted laparoscopic surgery to repair a uterine niche between 2013 and 2023. Before surgery, all patients were assessed using ultrasound imaging to check the thickness of the uterine wall (residual myometrial thickness, or RMT, adjacent myometrial thickness, or AMT and their ratio, called healing ratio, or HR) and to examine the size and shape of the niche. During surgery, doctors used the DaVinci robotic system to remove the niche and then stitched the uterine wall back together in two layers. After surgery, imaging showed that the uterine wall became significantly thicker, going from an average of 1.75 mm to 4.9 mm. Most patients (71.4%) reported that their symptoms improved or disappeared after surgery. Among the women who wanted to get pregnant, 65.4% successfully gave birth after the procedure. The study concluded that robotic-assisted surgery is a promising option for women with uterine niches, especially for those experiencing symptoms or struggling with fertility. However, more research is needed to develop treatment guidelines and to understand the long-term outcomes.

## Introduction

A uterine niche, or isthmocele, is defined as a myometrial defect at the site of a caesarean section with a depth of at least 2 mm.^1^ Isthmoceles occur in up to 60% of patients who have previously had a caesarean section and are symptomatic in about 30%–40% of cases.^2,3^ Sonographic detection depends on the technique used and the experience of the examiner.^4^ Performing sonohysterography with gel-/saline-instillation improves the detection rate from 56% to 84%.^3–5^

Various factors favour the development of uterine niches. These include a more caudal uterotomy^6,7^ and suturing technique.^8^ Current data suggest that double-layer suturing may be more beneficial compared to single-layer suturing.^8^ Locked sutures lead to more tissue ischaemia and therefore a thinner myometrium with an increased risk of suboptimal wound healing.^9,10^ Second-stage caesarean sections with lower incisions are particularly risky for the development of isthmoceles.^11^

Uterine niches are often overlooked in daily clinical practice; nevertheless, causing significant symptoms and complications in further pregnancies.^12,13^ The risk of uterine rupture, pregnancy implantation in the uterine scar (caesarean section pregnancy (CSP)), and placental abnormalities (placenta accreta spectrum (PAS)) in further pregnancies is increased in the presence of uterine niches.^13^ A myometrial thickness in the area of the CSP of <2 mm, determined during the first-trimester ultrasound, is associated with a greatly increased risk of developing PAS at birth.^14^

The correct sonographic measurement is important to allow tailored treatment planning according to the specific characteristics of the uterine niche, including niche length and depth, residual myometrial thickness (RMT) and adjacent myometrial thickness (AMT) in the sagittal plane, and niche width in the transverse plane.^1^ Typical symptoms are dysfunctional uterine bleeding (DUB), dysmenorrhoea and infertility.^13^ Obstetrical risks are also increased, especially in the case of RMT <2mm.^14^

There is disagreement in the current medical literature regarding the efficacy and benefits of surgical therapy in the treatment of niches.^2,3,15^ Some studies emphasise the potential benefits of surgical interventions by suggesting significant improvement in symptoms, but clear data in asymptomatic cases are lacking.^2^

In recent years, robot-assisted laparoscopic repair of uterine niches has become established as a treatment method at our tertiary centre. The aim of this study is to analyse robotic-assisted laparoscopic scar repair as a treatment modality of isthmoceles and to investigate the effects of the procedure on gynaecological symptoms and subsequent pregnancies.

## Materials and methods

### Study design and setting

This was a retrospective, monocentric observational cohort study conducted at a tertiary university hospital (Essen University Hospital, Germany). The study period comprised all consecutive robotic-assisted laparoscopic uterine niche repairs performed between 1 January 2013 and 31 December 2023. The report follows STROBE guidance for observational studies, including an explicit cohort flow and analysis sets.

### Population

Patients referred to our department with symptomatic uterine niches or complex niches (with multiple branches) as well as thin residual myometrium (<2 mm), and the desire to have children were informed about the option of surgical repair using robotic-assisted laparoscopy. A retrospective analysis of patients who had undergone robotic-assisted laparoscopic caesarean scar repair between January 2013 and December 2023 was performed. Patients were identified using a systematic search for the Diagnosis-Related Groups code N85.a (Isthmocele) and Operation and Procedure Codes 5-681.3 (Excision of other diseased tissue of the uterus) and 5-987 (Use of a surgical robot) in the hospital’s own information system. Included were premenopausal patients with a history of at least one caesarean section, diagnosed with symptomatic or complex uterine niche and who underwent robotic-assisted laparoscopic repair.

Patient characteristics such as age at the time of surgery, body mass index (BMI) and pre- and postoperative haemoglobin and haematocrit levels were recorded. Other parameters were skin-to-skin time, intra- and postoperative complications.

Patients were counselled for robotic repair if they met at least one of the following:

(i) *Postmenstrual spotting/abnormal uterine bleeding:* ⩾2 days of spotting after cessation of menses for ⩾3 consecutive cycles as documented in the chart;
(ii) *Pelvic pain/dysmenorrhoea:* persistent symptoms attributed to the niche after exclusion of alternative causes, documented as clinically relevant by the treating physician;
(iii) *Subfertility/infertility:* failure to conceive after ⩾12 months of unprotected intercourse (or ⩾6 months if age ⩾35 years), or referral for fertility evaluation with a niche considered as a main contributory factor;
(iv) *Thin residual myometrium:* RMT <2 mm on standardised transvaginal ultrasound;
(v) *Complex niche morphology:* >1 branching tract on contrast sonography as defined below.

Patients failing to meet the selection criteria or declining surgical intervention were excluded from the study. Selection into surgery was described descriptively and acknowledged as a limitation of the study.

Sonographic features (with and without contrast agent – ExEm® Foam; Killateeaun, Tourmakeady, Co. Mayo, Ireland) before and after surgery were also analysed. Postoperative symptoms and the outcome of subsequent pregnancies were recorded by means of telephone or personal interviews. Symptom outcomes were assessed by structured chart review supplemented by telephone and/or face-to-face follow-up where necessary. For verification, postoperative symptom status was classified using a pre-specified rubric: resolved (complete disappearance of the presenting symptom), improved (clear reduction in frequency/severity), unchanged, or worse. When documentation was insufficient to classify improvement conservatively, the outcome was documented as unchanged.

Patient stratification was based on the following pre-specified analysis sets: (1) *Primary cohort*: all eligible robotic-assisted repairs during the study period. (2) *Imaging analysis set*: participants with both preoperative and first postoperative standardised ultrasound measurements available. (3) *Symptom analysis set*: participants with documented preoperative symptoms and postoperative symptom assessment according to the rubric below. (4) *Fertility analysis set*: participants who reported desiring conception after surgery and had documentation of conception status and/or pregnancy outcome.

### Preoperative diagnostics

**Figure 1.**
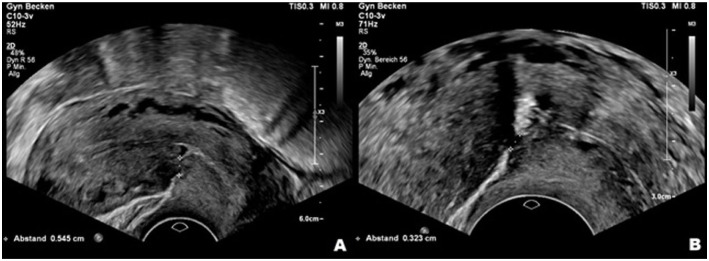
Sonographic evaluation of the residual myometrium thickness before and after contrast medium installation, showing a significant difference in the measured values. (a) Before contrast medium installation. (b) After contrast medium installation.

Patients were examined preoperatively using transvaginal ultrasound and hysterosalpingo-foam ultrasonography with ExEm® Foam to assess the size of the niches and the thickness of the overlying myometrium (Figure 1). The first postoperative assessment was defined as the earliest postoperative scan within a pre-specified window (preferably within 8–12 weeks). If multiple scans were available, the first within the window was used for the primary endpoint.

**Figure 2.**
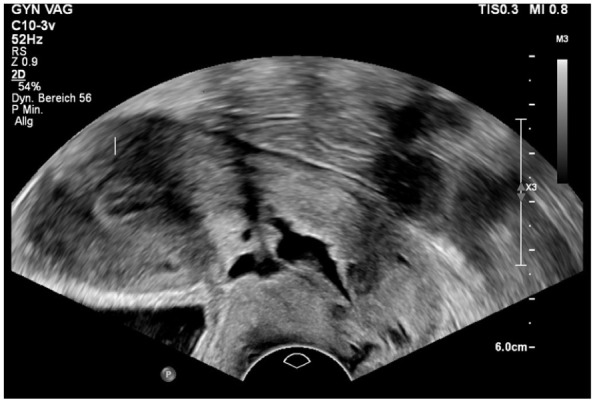
Transvaginal sonographic finding of a complex isthmocele with multiple branching tracts extending from the caesarean scar site.

The niches were classified as simple niche, without or with one branch, and complex niche, with more than one branch^1^ (Table 1, Figure 2). A branch was defined as a thinner part of the main niche that is directed towards the serosa and has a smaller width than the main niche.^1^ The RMT was measured as the smallest myometrial thickness from the niche to the uterine serosa in the sagittal plane and compared with the AMT, measured beside the niche, perpendicular to the cervical canal, where the myometrium is thickest.

**Table 1.** Preoperative patient characteristics.

Patient demographics	*N* = 51
Age (years)	33.65 ± 3.472
Body mass index (kg/m^2^)	25.7 ± 4.87
Gravida/Para	2/1
Number of previous C-sections (*n*)	
1	37 (72.5%)
2	9 (17.6%)
⩾3	5 (9.8%)
Uterus alignment, *n* (%)	
Anteversio	26 (51%)
Retroversio	17 (33.3%)
Centred	1 (2%)
Unknown	7 (13.7%)
Niche classification	
Simple	25 (49%)
Complex	25 (49%)
Scar pregnancy	1 (2%)
Preoperative symptoms	
Dysfunctional uterine bleeding	12 (23.5%)
Vaginal spotting	22 (43.1%)
Dysmenorrhoea	13 (25.5%)
Lower abdominal pain (inkl. Dyspareunia)	15 (29.4%)
Secondary infertility	20 (39.2%)

Measurements (RMT, AMT, niche size) were performed using electronic calipers on stored images. A single experienced reader performed the measurements. Inter- and intra-observer reliability were not assessed. The reader was blinded to clinical outcomes and other clinical data during measurement.

### Surgical technique

**Figure 3.**
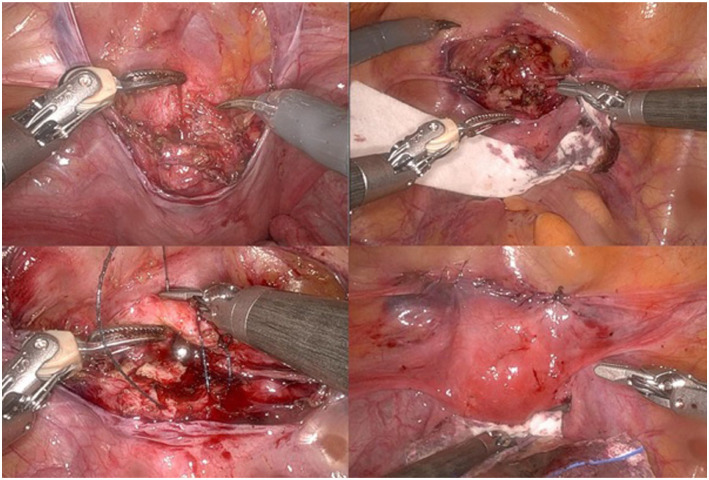
Intraoperative images from a robotic-assisted laparoscopic scar repair. (a; top left) Visualisation of the uterine niche after opening the uterine serosa. (b; top right) Excision of the niche. (c; bottom left) Closure of the uterotomy with V-Loc® sutures. (d; bottom right) Surgical site after completion of the operation.

Diagnostic hysteroscopy is performed before switching to robot-assisted laparoscopy to properly assess the characteristics of the uterine niche (isthmocele) from the uterine cavity. Robot-assisted laparoscopy is performed by experienced gynaecological surgeons at our centre using the Da Vinci-SI (until 2014) and XI (from 2014) (Intuitive Surgical, Sunnyvale, CA, USA). After attaching the trocars and docking (connecting the trocars to the patient-side cart), the surgeon starts the procedure at the console. The plica vesicouterina is carefully opened and the bladder is separated from the anterior wall of the uterus until the niche is visible. The entire tissue of the niche is resected with monopolar scissors. Healthy tissue around the dehiscence is then sutured in two layers from both wound angles using deep penetrating 2-0 V-Loc® sutures while sparing the endometrium. In the second row of sutures, the visceral peritoneum is closed again over the uterotomy (Figure 3). After hemostasis and removal of the specimens and instruments, the operation is completed.

After postoperative care, the patients were then discharged. A progestin-only pill to inhibit the production of cervical mucus and effectively prevent pregnancy was recommended for at least 3 months postoperatively from 2020. Postoperative sonographic monitoring was recommended and included hysterosalpingo-foam sonography with ExEm® Foam.

### Estimation of blood loss

Intraoperative measured estimated blood loss (EBL) based on the visual, pictographic or gravimetric methods was not routinely documented and was therefore not available for analysis.

**Figure 4.**
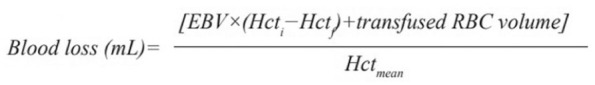
Lopez-Picado formula for the estimation of blood loss.^16^

The blood loss was estimated using the López-Picado formula,^16^ as it has been shown to be more consistent with directly measured blood loss than other blood volume loss formulas (Figure 4).

EBV is the estimated blood volume (determined according to Nadler, 1962^17^), Hcti is the preoperative haematocrit, Hctf is the postoperative haematocrit and Hctm is the mean haematocrit (between pre- and postoperative).

The blood volume was determined according to the Nadler formula^17^ for females as follows:

$$\begin{array}{l} \mathrm{Women}:\mathrm{Blood}\mathrm{volume}=\left(0.3561\times \mathrm{height}\left(m\right)3\right)\\ \;\;\;\;\;\;\;\;\;\;\;\;\;\;\;\;\;\;\;\;\;\;\;\;\;\;\;\;\;\;\;\;\;\;\;\;\;\;\;\;\;\;\;\;\;\;\;\;\;\;\;\;\;\;\;\;\;\;+\left(0.\mathrm{03308}\times \mathrm{weight}\left(\mathrm{kg}\right)\right)\\ \;\;\;\;\;\;\;\;\;\;\;\;\;\;\;\;\;\;\;\;\;\;\;\;\;\;\;\;\;\;\;\;\;\;\;\;\;\;\;\;\;\;\;\;\;\;\;\;\;\;\;\;\;\;\;\;\;\;\;+0.1833 \end{array}$$

Patients with a positive haematocrit delta were excluded from the aforementioned blood loss estimation. Therefore, López-Picado–derived EBL was calculated for 49/51 patients.

### Endpoints and hierarchy

The primary endpoint was the change in residual myometrial thickness (ΔRMT, mm) from preoperative assessment to the first standardised postoperative ultrasound assessment.

Secondary endpoints included: (i) change in healing ratio (ΔHR) and niche size; (ii) symptom outcome per rubric (resolved/improved/unchanged/worse); (iii) perioperative outcomes (skin-to-skin time; haemoglobin/haematocrit change; transfusion); (iv) complications postoperatively; (v) persistence/recurrence requiring reoperation with imaging confirmation; and (vi) fertility outcomes among patients attempting conception postoperatively, reported with explicit denominators.

Obstetric safety outcomes were abstracted from available maternity records for postoperative pregnancies. Outcomes were reported per pregnancy, restricted to the subset with detailed obstetric documentation. In addition to delivery mode and gestational age at outcome, we recorded suspected complications with their subsequent obstetrical outcomes.

### Statistical analysis

All data were documented in anonymised form. The statistical analyses were performed using SPSS Statistics® Version 27 (IBM, Armonk, NY, USA). Continuous variables are reported as mean ± SD, and categorical variables as *n* (%).

For paired pre- versus postoperative imaging outcomes (e.g., RMT, AMT, healing ratio, niche size) in patients with both measurements available, differences were analysed using a paired *t*-test for approximately normally distributed differences or the Wilcoxon signed-rank test otherwise.

For between-group comparisons (independent samples), the *t*-test was used for normally distributed data and the Mann–Whitney *U*-test for non-normally distributed data. To assess relationships between preoperative clinical parameters and postoperative outcomes, regression analyses were performed as appropriate. Pearson correlation coefficients were calculated to determine the strength and significance of associations. 95% confidence intervals (CI) were calculated using the Wilson CI. All tests were two-sided, and *p*-values <0.05 were considered statistically significant.

### Reporting standards

**Figure 5.**
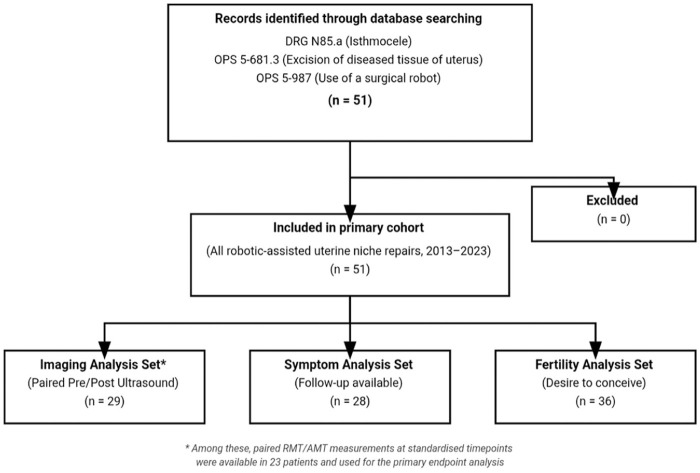
STROBE-flowchart of study design: all consecutive patients undergoing robotic-assisted laparoscopic uterine niche repair between 2013 and 2023 were included in the primary cohort (*n* = 51), with no exclusions. Analysis sets were defined and were availability-driven. Imaging analyses were restricted to patients with paired pre- and postoperative ultrasound measurements. Symptom outcome analyses included symptomatic patients with postoperative follow-up available. Fertility outcomes were assessed among patients with documented postoperative desire to conceive. STROBE, Strengthening the Reporting of Observational Studies in Epidemiology.

This study adheres to the Strengthening the Reporting of Observational Studies in Epidemiology (STROBE) guidelines for cohort studies to ensure transparency and completeness in reporting.^18^ A completed STROBE checklist is provided as a Supplemental File, indicating the location of each reporting item within the manuscript. The cohort structure and pre-specified analysis sets are depicted in Figure 5.

## Results

Fifty-one cases were identified who had undergone uterine niche repair using a Da Vinci Robotic System between 2013 and 2023 and were included in the primary cohort, with sets defined by data availability (Figure 5). The mean age of the women at the time of surgery was 33.6 ± 3.4 years (standard deviation, SD). The mean BMI was 25.7 (±4.87 SD) kg/m². Approximately 3/4 of the patients (72.5%) had a single C-section (CS) leading to niche formation and 17.5% had two or more CS (Table 1). In 17 (33.3%) patients, the last CS was planned, compared to 28 (54.9%) who had an unplanned caesarean section (11.8% unknown). The sonographic classification of isthmoceles was equally distributed: 25 patients (49%) had a simple niche, 25 (49%) had a complex niche and 1 patient had a scar pregnancy. A single-layer continuous uterotomy closure in the previous CS was present in 19.6% of the collective, a double-layer continuous closure in 15.7%, interrupted sutures in 2.0% and a continuous locking suture in 7.8%. Data about the closure of the uterotomy from 54.9% of patients was missing. The information on the last CS before the scar repair operation is recorded in Table 2.

**Table 2.** Data of the last CS.

Last CS	
Primary	17 (33.3%)
Secondary	28 (54.9%)
Unknown	6 (11.8%)
Uterotomy closure in the last CS	
Single-layer, continuous	10 (19.6%)
Double-layer, continuous	8 (15.7%)
Interrupted sutures	1 (2.0%)
Locked sutures, continuous	4 (7.8%)
Unknown	28 (54.9%)
Timing	
⩾37 week	29 (56.9%)
<37 week	6 (11.8%)
	16 (31.4%)

CS, C-section.

Data on preoperative symptoms were recorded from 49 women. Most patients were symptomatic preoperatively (39/49, 79.6%). The most common symptoms were vaginal spotting (43.1%), followed by secondary infertility (39.2%) and lower abdominal pain (29.4%). 20.4% of patients were asymptomatic preoperatively. The average operation time was 124.7 min. The López-Picado–derived EBL was 376.8 ± 183.4 mL (calculated in 49/51 patients; two patients with non-decreasing postoperative haematocrit [Hct_f ⩾ Hct_i] were excluded). Perioperative haemoglobin/haematocrit values and perioperative changes are presented in Table 3. No blood transfusions were administered. Complications were documented by review of peri- and postoperative encounters documented within 30 postoperative days according to the Clavien-Dindo classification, showing no intra- or postoperative complications within a 30-day postoperative timeframe.

**Table 3.** Perioperative haemoglobin/haematocrit values and perioperative change (pre − post) (Δ).

Variable	Mean ± SD	Median (IQR)	Min–Max
Preoperative Hb (g/dL)	12.99 ± 1.08	13.10 (12.60–13.70)	9.70–15.10
Postoperative Hb (g/dL)	11.86 ± 1.15	12.00 (11.20–12.50)	9.20–14.90
ΔHb (g/dL)	1.13 ± 0.63	1.20 (0.70–1.50)	−1.00–2.60
Preoperative Hct	0.386 ± 0.027	0.387 (0.372–0.406)	0.306–0.436
Postoperative Hct	0.355 ± 0.031	0.356 (0.340–0.376)	0.292–0.444
ΔHct	0.031 ± 0.019	0.036 (0.018–0.044)	−0.021–0.079

Postoperative haematocrit was non-decreasing in 2/51 patients; López-Picado–derived estimated blood loss was therefore calculated for 49/51 patients.

Nine patients (17.6%; 95% CI 9.6%–30.3%) underwent two operations because the niche persisted. Persistence was defined as persistent niche morphology on postoperative imaging with attributed symptoms. The interval from the primary surgery to repeat repair was 11.5 months (IQR 5–21). In three patients, the niche persisted despite two operations. Twenty-five patients (49%) were operated on after 2020 and received postoperative progestin therapy.

Our data showed no correlation between suturing technique, time or indication of the last caesarean section, BMI, nicotine abuse or secondary diseases with the incidence of isthmoceles.

In 23 patients, the RMT and AMT were measured preoperatively and postoperatively by ultrasound in our centre; an ultrasound check-up was made at a median of 12 weeks after surgery (IQR 11–13). The mean postoperative RMT was 4.9 ± 3.4 mm, compared to 1.75 ± 1.4 mm preoperatively (*p* < 0.001, 95% CI: −4.84 to −2.14). AMT remained relatively constant, from 10.6 ± 3.2 mm preoperatively to 10.7 ± 2.3 mm. Regarding the largest diameter of the niche, which was defined as the longest axis of the niche along the sagittal or coronal plane, the preoperative mean value was 11.3 ± 5.0 mm and postoperatively 9.1 ± 4.7 mm (*p* = 0.141). The calculated average HR pre- and postoperatively was 0.16 ± 0.14 mm and 0.39 ± 0.30 mm, respectively. This shows a markedly statistically significant increase in HR with (*t* = −4.55; standard deviation 0.28; confidence interval: lower −0.39/upper −0.14, *p* < 0.001).

A regression analysis was performed to examine the relationship between preoperative parameters and several symptoms among the study participants. After controlling for other variables in the model, DUB showed a marginally significant association with preoperative HR (β = 0.427, *p* = 0.052). However, spotting and pelvic pain did not exhibit statistically significant associations (β = −0.181, *p* = 0.375 for spotting; β = 0.303, *p* = 0.126 for pelvic pain). Notably, the presence of secondary dysmenorrhoea demonstrated a significant association with preoperative HR (β = −0.668, *p* = 0.013). Interestingly, RMT showed no statistically significant association with any preoperative symptom (Table 7).

Postoperative telephone and/or face-to-face interviews were conducted to fill in missing information of the patients who did not. The average follow-up time was 80.4 months. Postoperative symptom status was unavailable for 11/39 symptomatic patients. Of 28 patients who reported symptoms before surgery, 20 (71.4%, 95% CI 52.9%–84.7%) had an improvement: 11 (39.3%) of the 20 reported resolution of symptoms and 9 (32.1%) reported a reduction in symptoms. 8 (28.6%) of the symptomatic patients reported no change in gynaecological symptoms (Table 4).

**Table 4.** Postoperative outcomes.

Size of the niches	*N* = 29
Resolution	14 (48.3%)
Regression	14 (48.3%)
Unchanged	1 (3.4%)
Preoperative/Postoperative Mean niche size (largest diameter, mm)	11.3 ± 5.0/9.1 ± 4.7
Preoperative/Postoperative Mean of RMT (mm)	1.75 ± 1.4/4.9 ± 3.4
Symptoms	*N* = 28
Resolution	11 (39.3%)
Regression	9 (32.1%)
Unchanged	8 (28.6%)
Fertility outcomes^a^ (documented postoperative desire to conceive)	*N* = 36/51
Did not conceive	10/36 (27.8%)
Successful conception	26/36 (72.2%)
Conception, Abortion	5/26 (19.2%)
Conception, Birth	17/26 (65.4%)
Conception, currently pregnant	1/26 (3.8%)
Conception, outcome unknown	3/26 (11.5%)

a Denominators: conception status reported among *n* = 36; pregnancy outcomes reported among those who conceived (*n* = 26).

RMT, residual myometrial thickness.

Thirty-six patients expressed their desire to conceive postoperatively. Of these, 10 patients (27.8%) had not yet conceived at the time of follow-up, while 26 (72.2%, 95% CI 56.0%–84.2%) conceived. Among the 26 patients who conceived, 17 (65.4%, 95% CI 46.2%–80.6%) had live births. Five had one or more miscarriages (19.2%), one patient was pregnant at the last follow-up (3.8%) and three (11.5%) had unknown obstetrical outcomes. One patient gave birth by means of a spontaneous vaginal delivery (Tables 5–7). Postoperative fertility outcomes are presented using explicit denominators (desiring conception: *n* = 36; conceived: *n* = 26). The postoperative data and results are shown in Table 4.

**Table 5.** Paired sample *T*-test between the preoperative and postoperative parameters.

Variable	Mean	Std. deviation	Std. error mean	95% Confidence interval of the difference		*T*	One-sided *p*
				Lower	Upper		
Largest diameter	1.76875	6.33464	1.58366	−1.60674	5.14424	1.117	0.141
RMT	−3.51391	3.15851	0.65859	−4.87975	−2.1480	−5.335	<0.001
AMT	−0.22174	3.13861	0.65445	−1.57898	1.13550	−0.339	0.369

AMT, adjacent myometrial thickness; RMT, residual myometrial thickness.

**Table 6.** Regression analysis for association of preoperative symptoms with preoperative healing ratio.

Variable	Unstandardised coefficients		Standardised coefficients	Significance
	*B*	Std. error	β	
(Constant)	0.182	0.057		0.003
Dysfunctional uterine bleeding	0.089	0.045	0.427	0.052
Vaginal spotting	−0.037	0.041	−0.181	0.375
Secondary dysmenorrhoea	−0.134	0.051	−0.668	0.013
Pelvic pain	0.067	0.043	0.303	0.126

Dependent variable: preoperative healing ratio.

**Table 7.** Regression analysis for association of preoperative symptoms with preoperative residual myometrial thickness.

Variable	Unstandardised coefficients		Standardised coefficients	Significance
	*B*	Std. error	β	
Constant	1.645	0.337		<0.001
DUB	−0.015	0.590	−0.005	0.980
Spotting	0.094	0.478	0.035	0.845
Secondary dysmenorrhoea	0.960	0.604	0.310	0.122
Pelvic pain	−0.904	0.512	−0.302	0.087

Dependent variable: preoperative residual myometrial thickness.

DUB, dysfunctional uterine bleeding.

Detailed obstetric notes were available for a subset of postoperative pregnancies (*n* = 6) (Table 8). In the available records, no cases of caesarean scar pregnancy or placenta accreta spectrum were documented. One pregnancy underwent caesarean delivery at 36 weeks’ gestation for suspected uterine rupture due to progressive lower abdominal pain; intraoperative rupture was not confirmed. Three deliveries occurred preterm (31, 35 and 36 weeks of gestation), one pregnancy reached term, and one postpartum complication was documented (primary postpartum haemorrhage due to uterine atony). One early pregnancy event was recorded at 7 weeks of gestation (threatened abortion), which resolved uneventfully. Delivery mode was a caesarean section in all cases.

**Table 8.** Detailed obstetric notes for a subset of postoperative pregnancies.

ID	Gestational age at outcome	Mode of delivery	Documented complication(s)	Safety outcomes (CSP/Previa/PAS/Rupture)	Relatedness to index operation
P1	31 weeks	CS	(1) Cervical insufficiency managed with cerclage (2) IUGR	Premature birth	(1) Probable (2) Unlikely
P2	36 weeks	CS	CS for suspected uterine rupture; intraoperative rupture not confirmed	Premature birth	Unlikely
P3	35 weeks	CS	PPROM with preterm labour	Premature birth	Probable
P4	Postpartum	CS	Primary postpartum haemorrhage (uterine atony)	No documented negative outcomes	Unlikely
P5	Term	CS	Cervical insufficiency managed with Arabin pessary	No documented negative outcomes	Probable
P6	7 weeks	CS	Threatened abortion	No document negative outcomes	Unlikely

CS, caesarean section; CSP, caesarean scar pregnancy; IUGR, intrauterine growth restriction; PAS, placenta accreta spectrum; PPROM, preterm pre-labour rupture of membranes.

## Discussion

The RMT increased significantly after the robot-assisted treatment, while the diameter of the isthmocele decreased, albeit not statistically significant. While isthmocele size has traditionally been regarded as a key determinant of symptom severity and treatment outcomes, recent evidence suggests that RMT offers superior clinical benefit and predictive accuracy in the management of uterine niche-related complications.^12^ Moreover, diminished RMT has been associated with a higher likelihood of adverse obstetric outcomes, such as scar dehiscence and uterine rupture, highlighting its prognostic value in guiding preconception, counselling and pregnancy management.^19,20^ However, RMT alone may not fully capture the structural recovery of the uterine wall. The healing ratio (HR) offers a proportional measure that adjusts for anatomical variability. In our study, HR improved significantly postoperatively, even in patients with persistently thin RMT (<3 mm), suggesting that HR may serve as a more reliable surrogate for evaluating myometrial integrity. Notably, regression analysis showed that secondary dysmenorrhoea had a significant association with HR (*p* = 0.013), while DUB approached statistical significance (*p* = 0.052), indicating possible clinical relevance that warrants further investigation in larger cohorts. Ludwin et al.^19^ conducted a systematic review elucidating the intricate relationship between isthmocele size, RMT and AMT, demonstrating that RMT serves as a more reliable predictor of symptom severity and treatment response compared to isthmocele size alone. This contrasts with our data, since RMT showed no statistically significant correlation with preoperative symptoms. Instead, our results revealed that secondary dysmenorrhoea and DUB correlated, yet were not statistically significant, with preoperative HR. While some associations of preoperative symptoms with HR did not reach conventional levels of statistical significance, this hints at a potential relationship within this patient population.

The estimated intraoperative blood loss in our cohort averaged 376.8 mL, which may initially appear elevated for a minimally invasive procedure. However, this value was derived using the López-Picado formula,^16^ which incorporates perioperative haematocrit changes, as mentioned in the materials and methods section. It is important to note that such estimations can be influenced by hemodilution effects due to intraoperative and postoperative fluid administration and intravascular fluid shifts, potentially overestimating true blood loss. Because measured EBL and perioperative fluids were not routinely documented, we cannot quantify haemodilution; therefore, Hb/Hct changes and transfusion rates are presented alongside the formula-derived estimate (Table 3). Importantly, no intra- or postoperative complications were observed, and no patient required transfusion, supporting the clinical safety and tolerability of the procedure despite the calculated volumes.

The question of whether surgical treatment of isthmoceles is justified and, if so, which method, as well as which patient population (based on sonographic criteria or depending on accompanying symptoms) should undergo surgical intervention for isthmoceles, is still controversial.^15^ Our study, focusing on robotic-assisted laparoscopic repair, demonstrated a significant improvement in RMT, HR and symptom resolution in 71.4% of symptomatic patients postoperatively. This aligns with findings from Vervoort et al.,^21^ who reported notable symptom relief following laparoscopic resection of larger isthmoceles, supporting the effectiveness of minimally invasive approaches in enhancing patients’ quality of life.

When comparing our results with He et al.,^22^ who investigated fertility outcomes after hysteroscopic isthmocele resection, a complementary narrative emerges. Their study indicated improved fertility rates relative to expectant management in patients with an RMT of over 2.5 mm. Although our research did not focus explicitly on fertility metrics, 26/36 (72.2%) of patients who desired conception postoperatively conceived during follow-up, suggesting that robotic-assisted techniques may similarly support reproductive health.

Xia et al.^23^ evaluated the effectiveness of transvaginal repair versus hysteroscopic resection, revealing that both methods led to significant symptom improvement, with transvaginal approaches offering advantages in recovery time. Zhu et al.^24^ focused on predictors of success in hysteroscopic resection, emphasising the importance of isthmocele size and RMT. Our study corroborates this by identifying the HR as a possible indicator for symptom relief, reinforcing its role in guiding preoperative assessment and treatment strategies. Our data and the above-cited studies suggest that women with significant symptoms could potentially benefit from any surgery, as could those who are unsuccessfully trying to conceive. Although obstetrical outcomes in our documented cohort didn’t show major negative outcomes, cervical insufficiency and premature birth were reported in three patients, which raises the question of isthmocervical integrity. These findings should be interpreted descriptively, as obstetric outcomes were available only for a limited subset and were derived from retrospective record review; nevertheless, the absence of confirmed rupture or PAS in the available data is reassuring.

In nine patients, repeat repair of the isthmocele was necessary. As all patients underwent hysteroscopy before or during the procedure, intraoperative visualisation of the defect was ensured, and incomplete identification of the isthmocele is unlikely to explain these cases. It remains unclear whether these defects represented true persistence of the original isthmocele or de novo development postoperatively, as imaging cannot definitively differentiate between the two. The only common factor among these patients was older age, which may indicate impaired myometrial healing capacity and thus a predisposition to persistence or recurrence. Advanced maternal age has previously been described as a risk factor for isthmocele formation and impaired caesarean scar healing,^25,26^ supporting this observation in our cohort.

Within the IDEAL framework, this study is best positioned between Stage 2b (Exploration) and early Stage 3 (Assessment). It represents a retrospective, single-centre study of an operative technique in routine practice, with structured reporting of anatomical, clinical and reproductive outcomes, but without comparative effectiveness testing. Accordingly, the findings should be interpreted as hypothesis-generating and practice-informing within a treated population, supporting the need for future prospective studies.

### Limitations

Some limitations of our study should be noted. A formal sample size calculation was not performed due to the retrospective design of the study. The sample was based on all eligible cases over a 10-year period, limiting the ability to predetermine group sizes. While this restricts statistical power assessment, it reflects real-world clinical experience. Future prospective studies should incorporate formal power calculations to confirm these findings. In addition, the use of the DaVinci robotic system® is associated with increased costs, which not all centres/hospitals, depending on the health system of different countries, could endure.^27^ Not all patients were examined at the same phase of the menstrual cycle, and postoperative hormonal treatment was not standardised during the entire examined period. However, the study also reflects a learning curve in surgery as well as in perioperative management. Although 25 patients received postoperative progestin-only pill (POP) therapy after 2020, our study design and sample size did not allow for meaningful subgroup analysis of its effect on RMT or isthmocele size. It is biologically plausible that POP treatment may reduce endometrial proliferation, local inflammation and cervical mucous production, thus probably reducing the rate of recurrence of isthmocele formation.^25^ However, this hypothesis requires prospective validation in larger cohorts. Moreover, the estimated calculated blood loss was quite high, which could be attributed to postoperative haematocrit drop due to intraoperative and postoperative fluid therapy, which can mimic intravascular depletion due to blood loss.

Obstetrical outcomes were documented, in most cases, as categorical outcomes without specific dates (Conception, delivery, or last follow-up). This limitation prevented the use of time-to-event (Kaplan–Meier) analyses in the current dataset. Furthermore, outcomes were derived from retrospective chart review and were available for a small collective of patients; therefore, rare events may be underreported.

The failure rate of surgical therapy should also be considered: 27.8% of patients did not yet successfully conceive, and in 28.6%, surgery did not improve symptoms. Nevertheless, we analysed a cohort of consecutive patients that is consistent with and representative of actual clinical practice.

The strength of our study lies in its innovative approach, utilising modern Da Vinci technology and combining sonographic and surgical characteristics of the patients without neglecting the clinical aspects and fertility outcomes of the patients. The selection of suitable patients for surgical therapy requires careful evaluation.

## Conclusion

In conclusion, our findings suggest that robotic-assisted laparoscopic repair of isthmoceles is a safe and effective treatment option, particularly for symptomatic patients with DUB or secondary dysmenorrhoea. In addition, women with a desire for future fertility may benefit from surgical intervention in the presence of an isthmocele. These results support the role of minimally invasive isthmocele repair in carefully selected patients, with further prospective studies needed to validate long-term outcomes and refine patient selection criteria.

## Supplemental Material

sj-docx-1-reh-10.1177_26334941261426108 – Supplemental material for Robotic-assisted laparoscopic repair of isthmoceles: the feasibility of operative treatment and recommendations for patient selection Supplemental material, sj-docx-1-reh-10.1177_26334941261426108 for Robotic-assisted laparoscopic repair of isthmoceles: the feasibility of operative treatment and recommendations for patient selection by Sa’ed Almasarweh, Rainer Kimmig, Anna Magdalena Jakob, Anika Hüser, Paul Buderath, Roland Csorba, Angela Köninger and Antonella Iannaccone in Therapeutic Advances in Reproductive Health

## Declarations

**Ethics approval and consent to participate:** This study was approved by the Ethics Committee of the University of Duisburg-Essen (Approval number: 20-9353-BO). The requirement for informed consent was waived by the same committee due to the retrospective design of the study.

**Consent for publication:** Not applicable.

**Availability of data and materials:** An anonymised dataset underlying the analyses in this study, together with the statistical analysis, is available from the corresponding author upon reasonable request for purposes of independent verification. Access is subject to institutional data protection regulations and ethics approval constraints. A detailed data and analysis code can be shared to enable reproducibility of all reported results.
